# Editorial: Emerging Therapeutic Approaches for Repair and Regeneration of Injuries in the Peripheral Nervous System

**DOI:** 10.3389/fbioe.2022.891459

**Published:** 2022-03-31

**Authors:** Antonio Salgado, Xavier Navarro, Srinivas Madduri

**Affiliations:** ^1^ School of Medicine, University of Minho, Braga, Portugal; ^2^ Department of Cell Biology, Universitat Autònoma de Barcelona, Barcelona, Spain; ^3^ Department of Biomedical Engineering, University of Basel, Basel, Switzerland; ^4^ Department of Surgery, University of Geneva, Geneva, Switzerland

**Keywords:** nerve injury, cell therapy, nerve bioengineering, neurotrophic factors, nerve guidance graft, nano materials, growth factors delivery

The research topic entitled “Bio-systems engineering for regulation of nerve regeneration” is focused on new and emerging approaches for promoting nerve regeneration.

Acute and chronic nerve injuries often result in the loss of sensory, motor and autonomic functions with variable pathological changes arising from traumatic or non-traumatic forms of injuries. Despite the severe consequences of such injuries for the patients, existing treatment options, i.e., surgical repair, often using autologous nerve grafting, and pharmacological compounds, such as corticosteroids, non-steroidal anti-inflammatory and analgesic drugs, are all associated with important drawbacks and are far from being optimal solutions. Consequently, the quality of patient’s life is poor and the resulting socioeconomic impact is huge. Thus, there is a great need for further research to advance with new and innovative treatment strategies for improving functional recovery and patient wellbeing and to minimize the socioeconomic burden.

So far, research over the past 100 years has achieved remarkable achievements in the field of nerve regeneration, resulting in the advancement of micro-surgical nerve management, nerve cellular and molecular biology, nerve guidance scaffolds, cell therapy and nerve tissue engineering. Nevertheless, the existing knowledge and translational modalities still remain inadequate for clinical applications. Within this context, the collected research articles in this issue represent the work of 62 different authors in the form of three review articles and six original research reports, presenting new and emerging strategies involving interdisciplinary efforts ranging from cell and molecular biology, engineered nano-micromaterials, bio-active delivery, cell and gene therapy, tissue engineered grafts, and complex nerve conduit scaffolds. Taken together, the collected research reports strengthen existing knowledge in the field and provide further insights to move forward.

Immediately after traumatic nerve injury, Wallerian degeneration (WD) begins and Schwann cells play a key role in the subsequent cellular and molecular changes paving the way for axonal regrowth. However, there are no reported models for investigating the potential therapeutic interventions for regulating WD. Elsayed et al., report the establishment and characterization of an *in vitro* WD model, which would enable further research for basic as well as translational applications. Furthermore, their data revealed the beneficial effects of the adipose stem cells as evidenced by the upregulation of neurotrophic factors (NTFs) and CJUN, which are closely associated with the axonal growth program.

In continuation with WD, topographical guidance in the form of physical, biological and electrical signals guide the axonal growth and elongation towards the target organ. To address these requirements, Yu et al., Castano et al., and Ferrari et al. report on the beneficial effects of micro/nano-patterned functional surfaces and nerve cuff electrodes, respectively. Micropatterned poly (D,L-lactide-co-caprolactone) films functionalized with KHI peptide and nerve growth factor promoted axo-glial growth and *in vivo* nerve conduction to a significant level. Furthermore, PLA nanofiber with a 950 nm range size and SDF-1/CXCL 12 functionalities demonstrated the potential to overcome the inhibitory substrates and enhance the growth and migration of olfactory ensheathing cells. Interestingly, conductive polymer printed regenerative nerve cuff electrodes optimized with cost-effective fabrication technology resulted in enhanced motor axonal regeneration and function in rats after 3 months. In brief, materials engineered with micro/nano patterns and bio-chemical physical functions hold great promise for further developments aiming for the effective regulation of axonal elongation over long-gap (i.e., 30–60 mm) nerve injuries and for neuroprostheses.

In their work, Panzer et al. describe the fabrication of bands of Büngner in the form of tissue engineered micro-tissue. For this, hydrogel-based micro-columns with an inner diameter of 300 μm and an outer diameter of 700 μm were used for inducing the Schwan cells into dense and aligned cellular structures resembling bands of Bünger. Such a promising tissue-engineered approach will enable future developments towards off-the-shelf production of the aligned micro-tissue. Indeed, one of the major barriers for the production of off-the-shelf bio-based products is existing culture conditions, such as xenogeneic serum supplementation. As a potential solution to this problem, Guiotto et al. report on the use of human platelet lysate for the culture of adipose stem cell and further proved the synergistic function of platelet lysate in combination with extracellular matrix components for nerve tissue engineering applications. These results open up a viable and new option for the personalization of cell-based therapeutic product development.

Growth factors are widely accepted for their key role in regulating tissue growth and development. However, the current lack of appropriate delivery scaffolds and strategies has limited their therapeutic potential. In their review, Salvin et al. present the therapeutic potential of insulin-like growth factor-1, known for its trophic and protective effects on neuronal and non-neuronal glial cells. In addition, the review offers important insights on the most promising delivery routes and dosage forms for supporting nerve regeneration. Spinal root (ventral root) avulsion is critical and represents the most serious proximal nerve injury, given the fact that the distance between the lesion and target innervation site is too long and often results in a permanent loss of motor function. Among all the neurotrophic factors, glial cell line-derived neurotrophic factor (GDNF) is the most potent for the survival and growth of motor axons. In another review by Egger et al., recent advances in the field of GDNF gene therapy are presented, as well as future research directions emphasizing the importance of the spatio-temporal aspects of the therapeutic intervention, such as the location, timing, dose and duration of GDNF.

The extracellular matrix composition of nerve tissue is complex in nature and architecture. Biomaterials and bio-based research approaches are promising for recapitulating the key features and for instructing the sequence of cellular and molecular events of the axonal regeneration and nerve tissue formation. However, the detailed mechanisms underlying the interface regulation between engineered biomaterials and nerve tissue components are largely unclear. In a detailed review article, Powel et al. present such mechanisms with further information on the cell controllable micro-environment resulting from engineered bio-active biomaterials.

In summary, we consider that all the articles included in the current issue provide new and interesting data and important insights for further research in the field, particularly regarding the interdisciplinary aspects of nerve repair and regeneration approaches. The guest editors of this research topic thank all authors once again for their valuable contributions and hope that the readers in the field will be inspired by these significant research findings and expert opinions.

